# Trust Associated with South Korean Sojourners’ Chinese COVID-19 Vaccination Intent and Concerns: A Qualitative Study

**DOI:** 10.1007/s44197-023-00123-2

**Published:** 2023-06-01

**Authors:** Yujun Lin, Xiaoli Liu

**Affiliations:** 1 School of Journalism and Communication, Shandong University, Jinan, China; 2 School of Foreign Languages, Ludong University, Yantai, China

**Keywords:** South Korean sojourners, COVID-19 vaccination, Institutional trust, Media trust

## Abstract

People’s willingness to get vaccinated determines whether the campaigns against the COVID-19 pandemic can be successful in part. Considering the fact that both foreigners and its nationals are exposed to the risk of infection in China, the Chinese government has taken measures favorable to foreigners in terms of the vaccination, yet South Korean sojourners were reluctant to get China-developed COVID-19 vaccines. This study employed the trust in institutions and trust in media as a theoretical framework and seeks to analyze how these two affect South Korean sojourners’ intention to get Chinese COVID-19 vaccines. 25 South Korean sojourners living in Beijing participated in semi-structured interviews. The results showed that the mistrust South Korean sojourners have in China’s institutions and media, both traditional and social media, led to their reluctance to get Chinese COVID-19 vaccines. In addition, South Korean sojourners’ higher interpersonal trust in their peers also influenced their willingness to get vaccines. This study further interpreted such results from the perspective of cultural traits and national properties.

## Introduction

Over the past 2 years, the COVID-19 pandemic, as a global health emergency, has been exacerbating uncertainty and insecurity around the world. The major infectious disease also posed a great risk to sojourners. Sojourners refer to a group of people, including foreign students, foreign traders, diplomats, anthropologists doing research abroad, international journalists, and the like, who are willing to live in a country or region for some period of time out of their work arrangements or certain contracts [1]. As the pandemic became rampant, COVID-19 vaccines have been advocated as the most effective response against the SARS-COV-2 virus [2–4]. In this regard, the Chinese government gave mass vaccination priority and has been encouraging foreigners to receive the vaccination. On 26th March 2021, the Foreign Affairs Office of the People’s Government of Beijing Municipality released an announcement encouraging foreign nationals in Beijing to get vaccinated, voluntarily, of course. With valid certificates, such as a passport and residence certificate, foreign nationals who have joined Beijing’s social security and medical insurance service could receive free vaccination, while others would be charged CNY 93.5 per dose [5].

By the end of July 2021, more than 500,000 doses of Chinese vaccines have been administered to over 300,000 foreign nationals [6]. However, a poll in one report found that South Koreans showed the least willingness to get vaccinated: only 14% of respondents admitted that they wanted to receive the vaccination quickly [7]. In addition, more than half of South Korean diplomats in China were unwilling to receive Sinopharm or Sinovac COVID-19 vaccines (two China-developed vaccines) [8]. Despite the measures the Chinese government and health representatives have taken to advocate the benefits of receiving Chinese COVID-19 vaccines, the government still had to face South Korean sojourners’ vaccination hesitancy. Vaccination hesitancy refers to the phenomenon that individuals are reluctant or unwilling to receive certain vaccines, although the vaccines are available. It is a complex issue and varies depending on time, place, and the type of vaccine [9, 10]. Previous research showed that vaccine policies, cultural factors, levels of trust in institutions and media could affect individuals’ vaccination intention dramatically [11–15]. Therefore, the goal of this study is to investigate how trust in institutions and media affects South Korean sojourners’ vaccination intention of Chinese COVID-19 vaccines.

The primary contributions of the present study are as follows: (1) it contributes to a comprehensive understanding of South Korean sojourners under a national context; (2) trust in Chinese institutions and trust in China’s media are employed as a theoretical framework to explore South Korean sojourners’ vaccination willingness; (3) this study clarifies the correlation between South Korean sojourners’ reluctance to get China-developed COVID-19 vaccines and their trust in China’s institutions and media.

## Literature Review and Theoretical Background

### The Influence of Institutional Trust on Vaccination Intention

Previous research has found that three elements would determine the public’s trust in vaccination: knowledge and expertise; openness and honesty; and concern and care. For the public, apart from basic information and knowledge, they also want to make sure that the people who are conveying the information and clarifying concerns are trustworthy and their communication manner is acceptable [16]. Institutional trust refers to the assessment the public performs to measure whether risk management institutions (e.g., the government, the health systems, science, and the medical establishment) have the willingness or ability to help them avoid risks [17].

Some studies have tested the importance of institutional trust during the global pandemic across the globe. The more trust individuals show toward the institutions, the more willing they are to get relevant health services like the vaccination [18]. During the COVID-19 pandemic, people who trust their institutions can be more easily convinced by the official information in terms of the safety and efficacy of the vaccines, and therefore, they are more open to receiving the vaccine [19, 20]. Also, the public often relies on the vaccine’s country of origin to evaluate its safety and efficacy [21, 22]. A study revealed that in countries where people showed higher trust to a central government, including China, South Korea, and Singapore, the vaccination was more acceptable among the public [23]. On the contrary, lower trust in institutions always led to vaccination hesitancy [24]. Notably, the majority of people who have high institutional distrust were racial/ethnic minorities and populations having lower socioeconomic status; their life experience demonstrated that distrust often came from injustice, which ultimately made health inequality worse [25, 26]. Recent studies have found that excessive pro-research propaganda easily leads the public to believe the primary purpose of the government-encouraged mass vaccination was shifting from disease prevention to financial gains by healthcare providers and vaccine manufacturers, thus causing institutional distrust [27].

Many studies have found a link between institutional distrust, vaccine hesitancy and conspiracy beliefs. One study conducted in England demonstrated that people holding conspiracy beliefs showed less trust to guidelines released by the government as well as the institutions [28]. Interestingly, conspiracy beliefs tend to be enhanced by individuals’ behavior that only searches for and accepts information favoring their position and beliefs while ignoring unfavorable information [14]. In terms of vaccine hesitancy, conspiracy beliefs play a negative role in deteriorating one’s trust in the government, health system, and medical industry [29]. Empirical evidence has showed that conspiracy beliefs were associated with people’s perception of low socio-political control, politically-driven commands, and distrust in science [30–32]. Therefore, when people believed that the COVID-19 pandemic was driven by conspiracy beliefs, they were suspicious about the motivation behind every relevant measure put forward by the institutions, thus showing little willingness to get vaccinated [14].

### The Influence of Media Trust on Vaccination Intention

Media trust is another key factor regarding people’s health-related behavior, and may further influence the public’s health choices during a social crisis [33]. The information from different media channels varied according to their function and emphasis. Traditional media outlets, such as TV and newspapers, tended to provide more expert information [34]. During a health crisis when the public was in urgent need of authoritative information, traditional media outlets were regarded as a reliable source to both transmit knowledge and improve public awareness [35]. With the development of digital society, social media has been a breeding ground for health information regarding vaccines [36]. As an inclusive platform, social media, with vast unfiltered and latest information, was employed as a useful tool by the public [37]. Apart from media, interpersonal communication also mattered regarding health issues [38]. During a health crisis, interpersonal communication provided a network full of important information for people within it to communicate and deliver [39].

Extant research has shown that high trust in traditional media can significantly improve the public’s vaccination intention during a public health crisis. For example, in China, traditional media outlets, both national and local, were used as tools for encouraging the public to get vaccinated massively. Hence, the public’s high trust in traditional media would naturally contribute to lower vaccination hesitancy and higher vaccination willingness [40]. Surveys of nearly 2500 Americans during a measles outbreak demonstrated that users of traditional media were less likely to come across misinformation about the vaccines, thus showing higher vaccine acceptance [41]. Another study found that channels of information were used by the public to make vaccination decisions: individuals who received information from traditional media, such as national TV, national newspapers, and local newspapers, could receive more information about the vaccine, thus leading to a higher vaccination willingness [42].

Past studies have found that in addition to traditional media, people’s trust in social media played a vital role in their vaccination intention. The more trust people showed to social media, the more likely they were to get vaccinated [43]. Various sources of information, including websites and mobile applications, enhanced the public’s awareness of the virus, thus making the vaccination campaign easier to roll out [44–46]. For example, in terms of the HPV vaccination, the information from the media that advocated the benefits of HPV vaccines could help the public to understand the value, safety, and efficacy of those vaccines [47]. Nevertheless, several studies have demonstrated that social media was not an authoritative platform of health information, and in professional fields like vaccination, some information was even misleading [11, 48]. In terms of COVID-19 vaccination, unverified vaccine posts were ubiquitous, causing real damage to public health [49, 50].

Previous research has also shown that interpersonal communication among family members, friends and colleagues played a significant role in shaping one’s health intentions and behaviors [51, 52]. People having higher interpersonal trust were less likely to hesitate about terms of vaccination [53]. However, the dark side was that information from interpersonal communication was not always trustworthy because sometimes rumors might get a higher momentum than true information [54]. For example, a study found that the extremely high trust in interpersonal communication could affect people’s COVID-19 vaccination motivation in the future; specifically, the extremely frequent person-to-person communication might make it harder to roll out the vaccination campaign, because some people might find it special and eye-catching if they did not get vaccinated [40].

While studies have investigated the correlation between the public’s trust and their vaccination result, few of them have combined trust in institutions and trust in media as a theoretical framework to analyze their influences on people’s vaccination willingness. Studies that have explored the importance of trust in institutions or trust in media mainly focused on local residents, while few have examined how the sojourners’ trust in institutions and media influenced their vaccination willingness in the host country. Besides, no study has shed light on South Korean sojourners living in China in this respect. To address this research gap, this study proposes the following research questions:

1. RQ1: How does South Korean sojourners’ trust in China’s institutions influence their willingness to get China-developed COVID-19 vaccines?
2. RQ2: How does South Korean sojourners’ trust in China’s media influence their willingness to get China-developed COVID-19 vaccines?

## Materials and Methods

### Study Design

The purpose of this research is to investigate how South Korean sojourners’ trust in institutions and trust in media influence their intention to get Chinese COVID-19 vaccines. A qualitative research method was utilized to achieve research goals. Qualitative research can provide researchers a more unrestrained, profound, and more flexible understanding of the target group’s experiences, with higher levels of openness and the potential to adapt to changes as the inquiry goes deeper [55]. In terms of individuals’ vaccination willingness, qualitative studies are rare, and due to the exploratory nature of the current study that focuses on meaning-making [56], a qualitative design should be more appropriate to explore abundant and unconstrained information about this topic.

This study adopted one-to-one semi-structured interviews. Semi-structured interviews were appropriate because of its two features. First, to explore respondents’ perceptions of and attitudes toward complicated and even sensitive subjects and search for as much information as possible, semi-structured interviews are necessary. Second, considering the fact that respondents have different educational, professional and personal backgrounds, semi-structured interviews are more suitable than standardized interviews [57]. Therefore, with open-ended questions, the semi-structured interview is ideal to explore how trust in institutions and trust in media influence South Korean sojourners’ willingness to receive Chinese COVID-19 vaccines.

### Participants

This research focused on South Korean sojourners in Beijing. All respondents were informed of the purpose of this study, and they participated in semi-structured interviews voluntarily. The initial interview list was provided by the South Korean Chamber of Commerce in China, and the researcher conducted a purpose sampling, forming a preliminary interview list. Later, a snowball sampling was conducted to enlarge that list. The snowball sampling was based and expanded on the following selection criteria: all interviewees (a) have been living in Beijing for at least 1 year; (b) having South Korean nationality; (c) have gone through the COVID-19 pandemic; and (d) have not been infected. Finally, a list with 25 interviewees was formed. Among them, 10 were female and 15 were male. These interviewees were aged between 18 and 69 years old. Their sojourn period varied, and they had different levels of Chinese proficiency.

### In-depth Interviews

Since the outbreak of the COVID-19 pandemic, to defeat the virus as soon as possible, China has been imposing strict regulations, including wearing masks, keeping social distance, limiting big gatherings, and regulating travel. Against this background, peer-to-peer online in-depth semi-structured interviews were conducted, and each one of the 25 interviewees was interviewed for 1 h or so. The interviews began with basic questions (“How long have you been living in Beijing?; What’s your profession?; Have you been received Sinopharm or Sinovac COVID-19 vaccines?), and then moved to unstructured open-ended questions about South Korean sojourners’ viewpoints on the influence of Chinese institutions and China’s media on their vaccination intention.

During the interview, the researcher asked questions in Chinese, and interviewees could respond either in Chinese or Korean, whichever they were more comfortable with. Of course, to make sure that the two sides could understand each other well, another research team member, Dr. Liu, a university lecturer in Korean, was present in every interview. Dr. Liu majored in Korean translation, and she has been teaching Korean in China for more than 10 years, indicating that her assistance was credible. With the consent of the interviewees, every session was recorded.

### Data Analysis

Upon the completion of each interview, Dr. Liu would compile the recording data into transcripts, and then, both the transcripts and original recording would be sent to a professional translation firm to proofread, thus guaranteeing the accuracy of the transcripts. Later, the proofread version would be sent back to Dr. Liu for final check and confirmation to ensure consistency between the original interview content and the transcripts.

This research employed thematic content analysis to analyze all data from interviews. Thematic content analysis is a frequently used qualitative descriptive approach that requires the data analyst to immerse himself/herself into the data to see the whole picture [56, 58]. Therefore, it is the perfect choice for this study which analyzes a lot of interview data and categorizes them into different themes. Based on the research questions, we conducted a thematic analysis of the trust in institutions and trust in media based on interview transcripts. In terms of trust in media, a thematic analysis of traditional media, social media and interpersonal communication was conducted.

## Results

China was among the first countries that promoted nationwide vaccination, with foreign nationals included. After the COVID-19 vaccination campaign rolled out in Beijing in March 2021, different districts subsequently released notifications to inform foreign nationals of the latest vaccination information. However, despite the convenient services, all interviewees in this study reported no COVID-19 vaccination history of China-develop vaccines. Among the 25 participants, 5 who had been vaccinated received the vaccine in South Korea. Some unvaccinated interviewees mentioned that they would consider going back to South Korea to get COVID-19 vaccines if policies in China forced them to get vaccines and their normal life would be disrupted otherwise.

### Influence of Trust in Chinese Institutions

Nearly 80% of interviewees said that the over-hyping of COVID-19 vaccination campaigns by the Chinese government led to their doubt about the motivation behind it. Most of the respondents (*n = *22) thought that such regulations were out of the pursuit of political and economic interests. Interviewee 3, an employee in a Cafe, believed that although vaccination charges for each person were not that high, it would still be lucrative if foreigners in China all got vaccinated. Moreover, some interviewees (*n = *20) worried that the Chinese government would never make foreign nationals a priority and the latter might even be vaccinated with inferior vaccines. Interviewee 10, owner of a South Korean restaurant, claimed that foreign nationals might get different vaccines from what the Chinese people got considering the fact that it was hardly possible to produce a large number of vaccines in a short period of time. Even worse, some participants (*n = *16) believed that the COVID-19 vaccination campaign toward foreign nationals was a conspiracy. Interviewee 13, manager of a feed company, thought that the South Korean government’s refusal of Chinese COVID-19 vaccines could perfectly explain South Korean sojourners’ reluctance. Interviewee 20, sales executive of an electronic enterprise, mentioned, “The conspiracy theory has been ubiquitous since the outbreak of the COVID-19 pandemic. Honestly, I also suspect that there is some kind of conspiracy behind the mass vaccination campaigns, including the great support toward foreigners, advocated by the Chinese government.”

Many interviewees in this study expressed their concerns about vaccines developed by China. Therefore, they were reluctant to get Chinese COVID-19 vaccines. Some respondents (*n = *20) claimed that China’s vaccine R&D was too fast to be reliable. On one hand, they could not find much information on vaccines developed by China. On the other hand, they said that current vaccines had not gone through abundant clinical tests to prove their efficacy and possible side effects. Interviewee 22, a lawyer, worried that the current clinical tests could not deny future risks of the vaccination since Chinese scientists had developed those vaccines in a very short period of time without informing the public of the R&D process and ingredients of vaccines. Interviewee 17, a housewife doubted the efficacy of Chinese COVID-19 vaccines because of the sporadic outbreak of the virus now and then under the context of mass vaccination. Moreover, some interviewees (*n = *18) were uncertain about the capability of Chinese medical establishment to deal with adverse reactions of the vaccination. Interviewee 21, a HSK trainer, mentioned, “Vaccination always brings adverse reactions, such as headache, fever, secondary infection, and the like. I am not sure that China’s medical system is capable of solving these potential problems. China has been developing rapidly in recent years, but the fact is that China remains to be a developing country with limited medical capacity.”

### Influence of Trust in China’s Media

Over half of the participants said that they had no trust in the information from China’s media, indicating that China’s media outlets failed to persuade them to get vaccinated. Many participants (*n = *17) complained that the excessive promotion of the advantages of the COVID-19 vaccination by China’s traditional media, such as broadcasts and TV, added to their doubts. Interviewee 8, manager of a trade company, admitted that his doubts about China-developed COVID-19 vaccines came from various news on TV where many scientists and doctors were encouraging the vaccination. Interviewee 25, teacher of an international elementary school, mentioned that the overwhelming information advocating benefits of the vaccination on TV was far from persuasive because almost all vaccines, regardless of their type and function, could cause certain side effects. Moreover, other respondents (*n = *16) were suspicious about the vaccination information from China’s traditional media since they believed that those traditional media outlets were regulated and even controlled by the Chinese government. Interviewee 24, a housewife, thought that all information on TV programs in China reflected the government’s will. In her eyes, it was natural for Chinese people to trust the Chinese government, but South Koreans were inclined to trust their government and were suspicious about information in China’s TV programs.

Apart from traditional media, social media also influenced respondents’ attitudes toward the vaccination of China-developed COVID-19 vaccines. Misinformation went viral on Chinese social media, adding to South Korean sojourners’ sense of insecurity. Some respondents (*n = *18) admitted that they did not know which vaccination information was reliable on social media since they were flooded with vast amounts of information every day. Interviewee 5, manager of a machinery company, complained that the majority of information on social media remained unchecked, making him more anxious rather than helping him understand the virus and vaccine better. Interviewee 2, a Korean translator, believed that the information on China’s social media was not qualified to act as vaccination guidance. Besides, most of the interviewees (*n = *22) said that China’s social media platforms exaggerated the risks of not getting vaccinated, which was neither accurate nor convincing. Interviewee 1, a student said, “My Chinese fellows always forwarded me some information about the vaccination of China-developed vaccines, as well as reports of some foreign media praising China’s mass vaccination campaign, but I only regarded them as the over-hyping and fake information.”

Lastly, the interpersonal communication between South Korean sojourners and their South Korean peers also influenced their vaccination behaviors in China. Most respondents (*n = *16) claimed that the behaviors and suggestions of their peers affected their intention to get China-developed COVID-19 vaccines. According to interviewee 19, an IT engineer, he was unvaccinated because his South Korean friends in China expressed concerns about Chinese vaccines. Interviewee 15, a former employee in a supermarket, who had little contact with local Chinese and got most of the virus-related information from his South Korean friends, was unvaccinated because those friends regarded China’s mass vaccination campaigns as a conspiracy. Those respondents who have been vaccinated (*n = *5) all got their jabs in South Korea at the suggestion of their peers. Interviewee 11, a government representative, mentioned, “Due to the nature of my work, the unvaccination may cause considerable inconvenience. Therefore, I accepted the suggestion of my South Korean colleagues and got vaccinated back in South Korea.”

## Discussion

This study explored the impact of South Korean sojourners’ trust in institutions and trust in media on their vaccination intention as well as concerns about China-developed vaccines during the COVID-19 pandemic through qualitative research based on in-depth semi-structured interviews. After analyzing the interview data of 25 interviewees, it was clear that South Korean sojourners’ mistrust in Chinese institutions and China’s media lowered their willingness to get Chinese COVID-19 vaccines.

Consistent with previous studies [9, 10], South Korean sojourners did have vaccine hesitancy. The World Health Organization (WHO) declared vaccine hesitancy as one of the top ten global health threats. The high vaccine hesitancy might lead to the failure of immunization efforts. The current study found that the attitude of South Korean sojourners toward China-developed COVID-19 vaccines was even worse than vaccine hesitancy: they completely refused to receive Chinese vaccines. South Korean sojourners’ vaccine hesitancy related to their distrust of Chinese institutions, which verified the past research [24]. The high institutional trust was an important factor facilitating the public’s COVID-19 vaccination willingness, and institutional trust had the greatest impact on the public’s vaccination decision [14, 19, 20]. However, in this research, South Korean sojourners showed mistrust in Chinese institutions.

On one hand, South Korean sojourners distrust the Chinese government. Since the beginning of the mass COVID-19 vaccination campaign, the Chinese government has been mobilizing every sector in society to improve vaccination rates by educating the public about the vaccine’s safety and efficacy, disseminating information about the risk of the virus and non-vaccination, organizing vaccination programs, and reporting real-time vaccination progress, in the hope of convincing people to get vaccinated as soon as possible. Nevertheless, studies have reminded us that excessive pro-research propaganda easily leads the public to doubt the primary purpose of government-endorsed mass vaccination [27]. Most participants in this study thought that the Chinese government encouraged mass vaccination among its public and foreign nationals to pursue economic interests, and they even regarded it as a conspiracy. Conspiracy beliefs further led to South Korean sojourners’ vaccine hesitancy and exacerbated their distrust of the Chinese government.

On the other hand, South Korean sojourners were suspicious about the safety and efficacy of China-developed vaccines, indicating that they distrusted Chinese medical establishment and science. Those who had more trust in science were inclined to show positive attitudes toward the COVID-19 vaccination and were more willing to get vaccinated, while the distrust in science always led to vaccination hesitancy and negative attitudes toward vaccines [59, 60]. Also, as other researchers have noted [21, 22], the public relied on the vaccine’s country of origin to evaluate its safety and effectiveness. In this study, those five participants who have been vaccinated received the vaccines in South Korea, meaning that they trusted vaccines developed by their home country more. As for those participants who had no intention to get Chinese COVID-19 vaccines, their mistrust in China’s medical system and science was the main reason.

In this research, China’s media failed to improve South Koreans’ willingness to get China-developed COVID-19 vaccines, which was somewhat different from those of previous findings [33, 36]. The reason behind it was that the sojourners did not trust the information from traditional media in China. Most South Korean sojourners believed that traditional media outlets were controlled by the Chinese government and acted as the official mouthpiece. Therefore, they were suspicious about the vaccination information conveyed by China’s traditional media. In addition, the current study highlighted the influence of sojourners’ identity traits on their trust in traditional media in terms of the COVID-19 vaccination. Different from this finding, previous research demonstrated that as a high-credibility message source, traditional media could promote the vaccination campaign by reporting the potential risk of a certain virus [40–42]. In this study, however, for those South Korean sojourners who held no trust toward China’s institutions, traditional media, which represented the Chinese government, was no longer a highly credible information source.

The past literature has concluded that social media’s influence on vaccination intention is a double-edged sword. For one thing, social media helped to increase vaccination rates during the pandemic. The employment of social media, including text messaging, smartphone applications, targeted websites and portals, was effective in disseminating information, increasing vaccination intention, and improving vaccination uptake [44–46]. For another, misinformation and fake news were common on social media during the pandemic, which weakened its credibility and caused vaccine hesitancy [49, 50]. However, contrary to earlier studies, this study found that Chinese social media did not exert direct positive influence on South Korean sojourners’ vaccination intention. Vast amounts of information on social media platforms failed to educate South Korean sojourners about the importance of the COVID-19 vaccination; on the contrary, they did not trust the information on China’s social media. Moreover, many online comments and praise from major foreign news agencies on China’s vaccination efforts were regarded as delicately designed propaganda.

Media trust is one of the key factors influencing people’s health decisions and behaviors. Existing research has found that people who had a higher education level, who were married, and who had not been vaccinated against the SARS-COV-2 showed greater trust in traditional media, while people who had a higher income and had not been vaccinated showed greater trust in social media [40, 61]. However, the current study found that South Korean sojourners interviewed did not trust China’s traditional media nor social media even if some of the participants had received higher education, were married, had higher incomes, and had not been vaccinated. Interestingly, this study found that in terms of vaccination, South Korean sojourners showed more trust in interpersonal communication, which, of course, happened among South Koreans, not between South Korean sojourners and local Chinese. Most respondents perceived Chinese COVID-19 vaccines as unsafe and ineffective through communication with their peers, while those respondents who have been vaccinated all got information about the necessity of the vaccination from their peers and went back to South Korea to get their jabs. This finding conforms to the research results that interpersonal communication with families and friends could facilitate individuals’ vaccination behaviors [51–53], but diverges from those findings that attributed the lower vaccination rate to interpersonal communication [40, 54].

Notably, there is a deeper reason for South Koreans to mistrust the Chinese institutions and China’s media while showing absolute trust to the interpersonal communication among their peers–South Koreans’ unique national pride and their collectivism-oriented culture. Previous research pointed out that the racial minority usually showed greater mistrust to the government [25, 26]: and the vaccination willingness was influenced by cultural factors [15] with South Koreans having a high vaccination rate out of their high trust in a central government [23]. The finding of this study verified and expanded these results. In South Korea, national spirit education was a key part of its education system, and adolescents were imparted with patriotism and national pride from an early age. With a strong sense of national pride, South Koreans are quite loyal to their country and the government. Since the beginning of China’s vaccination campaign, South Korea has been rejecting China-developed vaccines. Then, naturally, South Korean government’s disapproval of Chinese vaccines has deteriorated South Korean sojourners’ trust in Chinese institutions. Additionally, South Koreans grow up in an environment advocating collectivism, which champions the belonging of an individual to a large group, such as a family, a religion, and a country, and emphasizes the importance of mutual communication and help among a group. Hence, South Korean sojourners showed great trust to their peers rather than China’s media, which further lowered their willingness to get China-developed vaccines.

## Conclusions

This study focused on the influence of the trust in institutions and trust in media on the willingness to get Chinese COVID-19 vaccines of South Korean sojourners living in Beijing. Through in-depth semi-structured interviews with 25 participants, this study found that South Korean sojourners hold an extremely low intention to get China-developed COVID-19 vaccines due to their mistrust in China’s institutions and media. Meanwhile, South Korean sojourners’ interpersonal communication with their peers also affected their vaccination willingness. Although the Chinese government encouraged foreign nationals to receive COVID-19 vaccines, South Korean sojourners showed low vaccination intention, and their choice to not get vaccinated in China added a burden to China’s pandemic prevention and control. Therefore, it is of great importance for the Chinese government and media to deal with the vaccination hesitancy of South Korean sojourners, which should also be the emphasis of relevant research in the future.

## Limitations

This study has the following limitations. First, the current study was conducted during a certain period of the pandemic, and it was possible that South Korean sojourners’ attitudes toward Chinese COVID-19 vaccines could change as the pandemic evolved. Second, this study focused on South Korean sojourners in Beijing, while those living in other cities in China were not covered, indicating that South Korean sojourners in other cities may have different levels of vaccination intention and concerns. Third, there is no discussion on specific measures that the Chinese government and media should take to enhance South Korean sojourners’ trust, which can be a starting point for later studies.

## Data Availability

Data are not publicly available because the information may compromise the privacy of research interviewees.
